# Supplementary material for: Improving Resolution and Resolvability of Single Particle CryoEM Structures using Gaussian Mixture Models
Source: ArXiv. 2023 Aug 29:arXiv:2303.18241v2. Preprint. [Version 2] (PMC10491338)
Supplement: 1 [file NIHPP2303.18241V2-supplement-1.pdf]

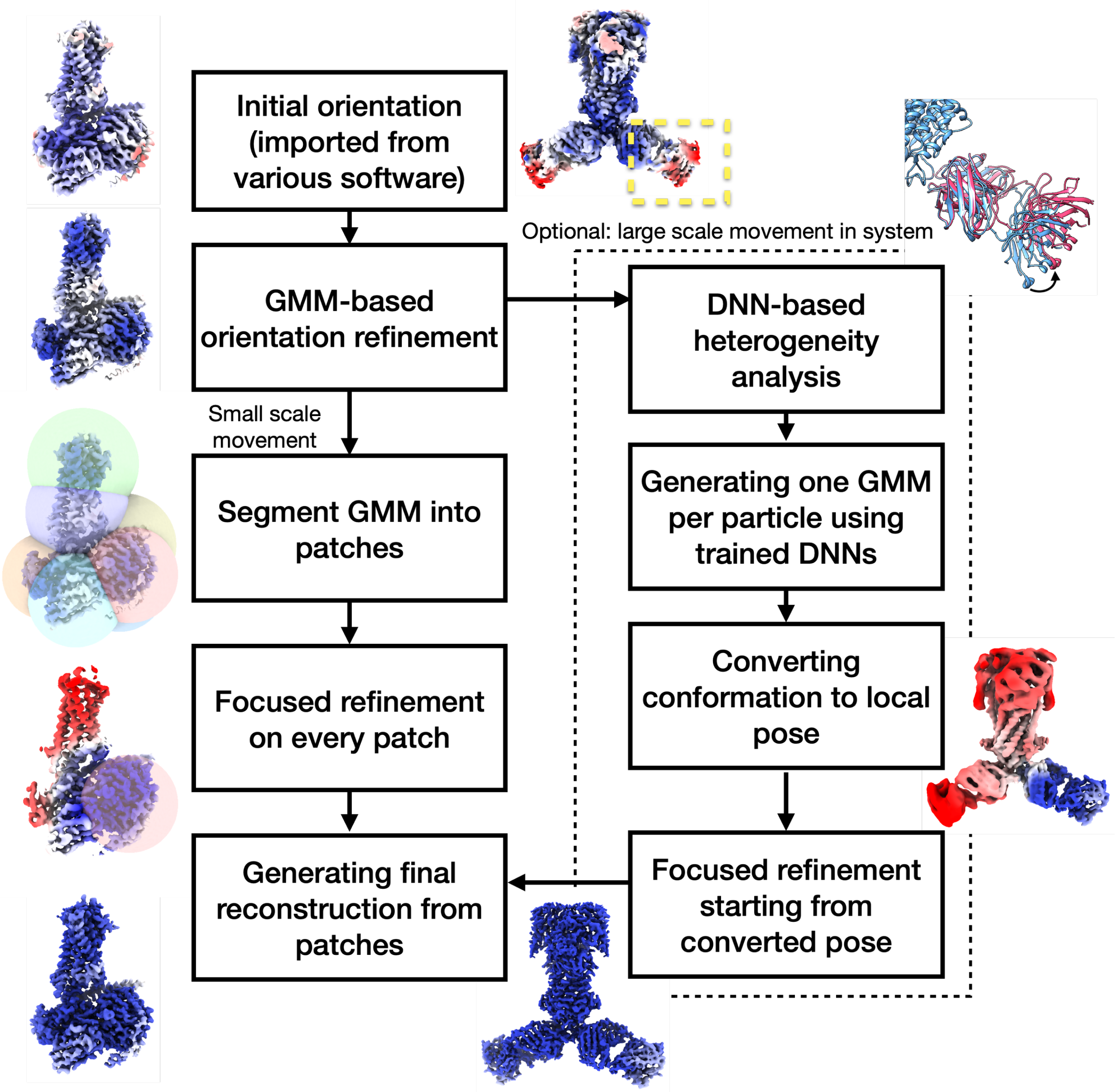

Fig S1. Workflow diagram for GMM-based particle orientation and conformation refinement. Each block represents one step of processing, and the arrows indicate the sequence of the processes. The right side, DNN-based heterogeneity analysis is optional and is only recommended when large scale movement is present in the system. The result of the DNN-based refinement focusing on one region can be treated as one patch and merged into the patch-by-patch refinement results from the main workflow.

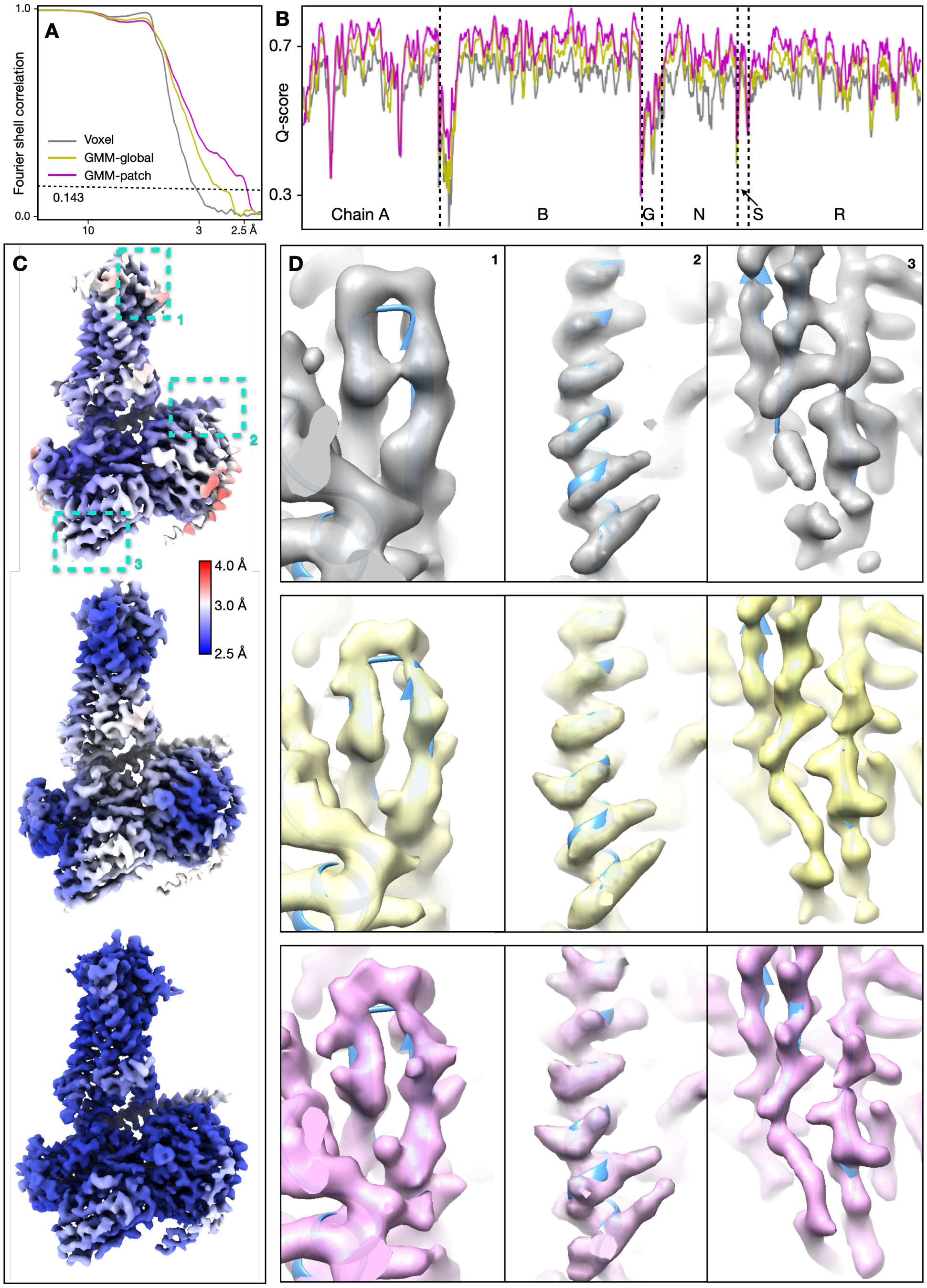

Fig S2. Detailed results of the GPCR dataset (EMPIAR-10786). (A) “Gold-standard” FSC curves of the reconstruction using initial orientation (gray), global GMM-based refinement (yellow) and patch-by-patch refinement (pink). (B) Q-score comparison of the three corresponding maps. (C-D) Overall structure of the three reconstructions as described in A and B, colored by local resolution, and comparison of local real space features. The cyan boxes in C highlight the location of features in the corresponding columns shown in D.

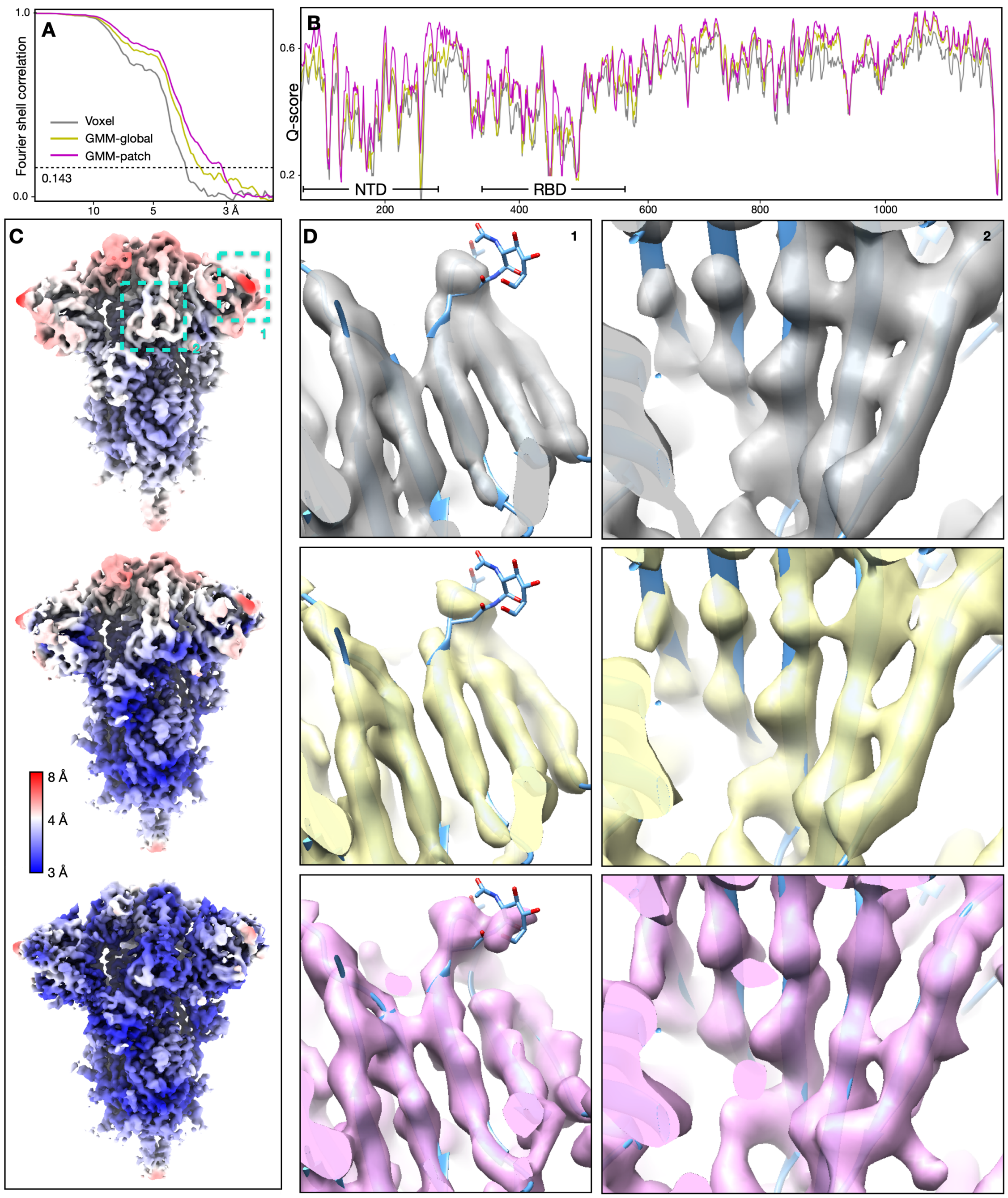

Fig S3. Detailed results of the SARS-COV2 dataset (EMPIAR-10492). (A) "Gold-standard" FSC curves of the reconstruction using initial orientation (gray), global GMM-based refinement (yellow) and patch-by-patch refinement (pink). (B) Q-score comparison of the three corresponding maps. (C-D) Overall structure of the three reconstructions, colored by local resolution, and comparison of local real space features. The cyan boxes in C highlight the location of features in the corresponding columns shown in D.

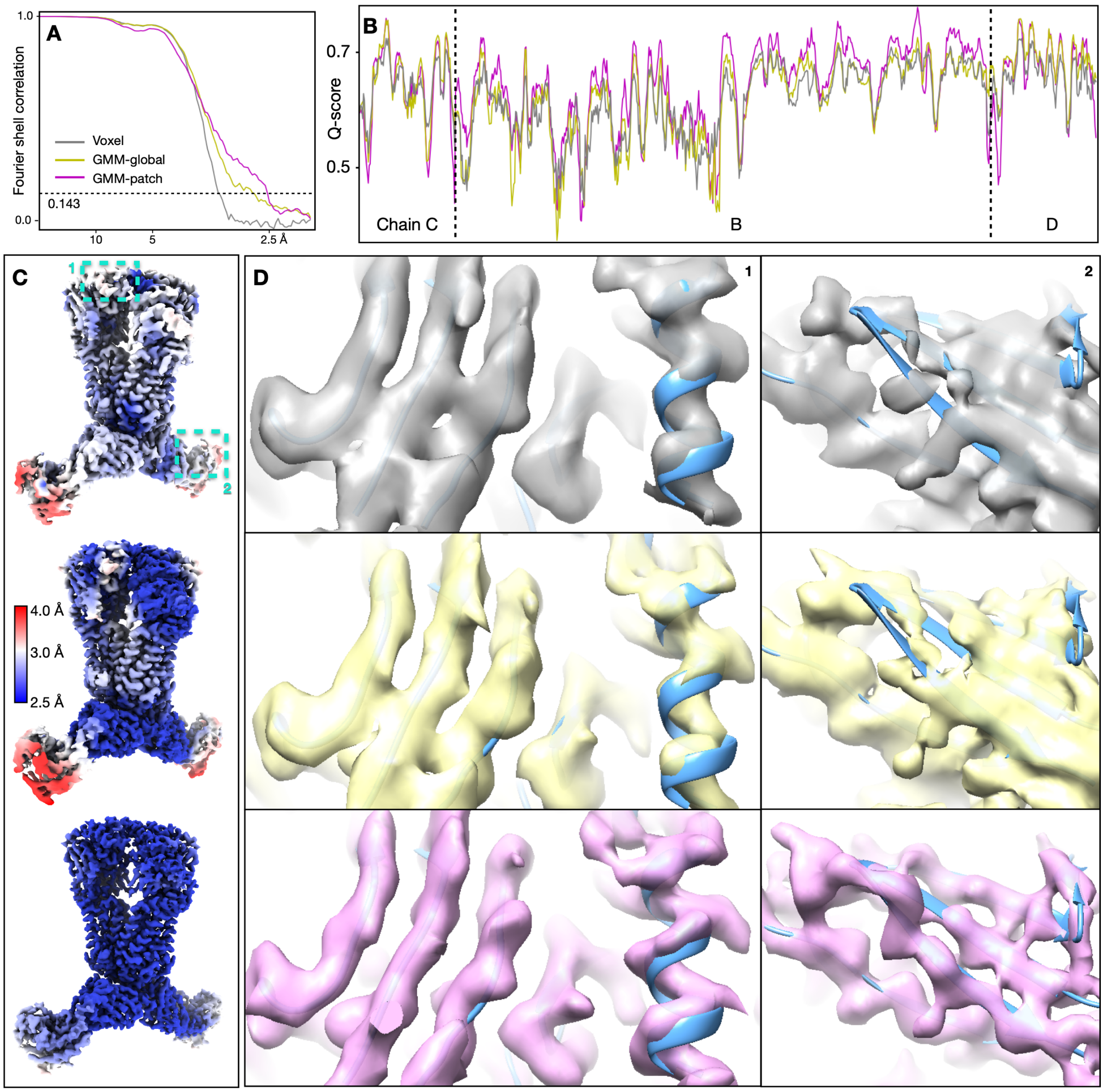

Fig S4. Detailed results of the ABC transporter dataset (EMPIAR-10374). (A) "Gold-standard" FSC curves of the reconstruction using initial orientation (gray), global GMM-based refinement (yellow) and patch-by-patch refinement (pink). (B) Q-score comparison of the three corresponding maps. (C-D) Overall structure of the three reconstructions, colored by local resolution, and comparison of local real space features. The cyan boxes in C highlight the location of features in the corresponding columns shown in D.

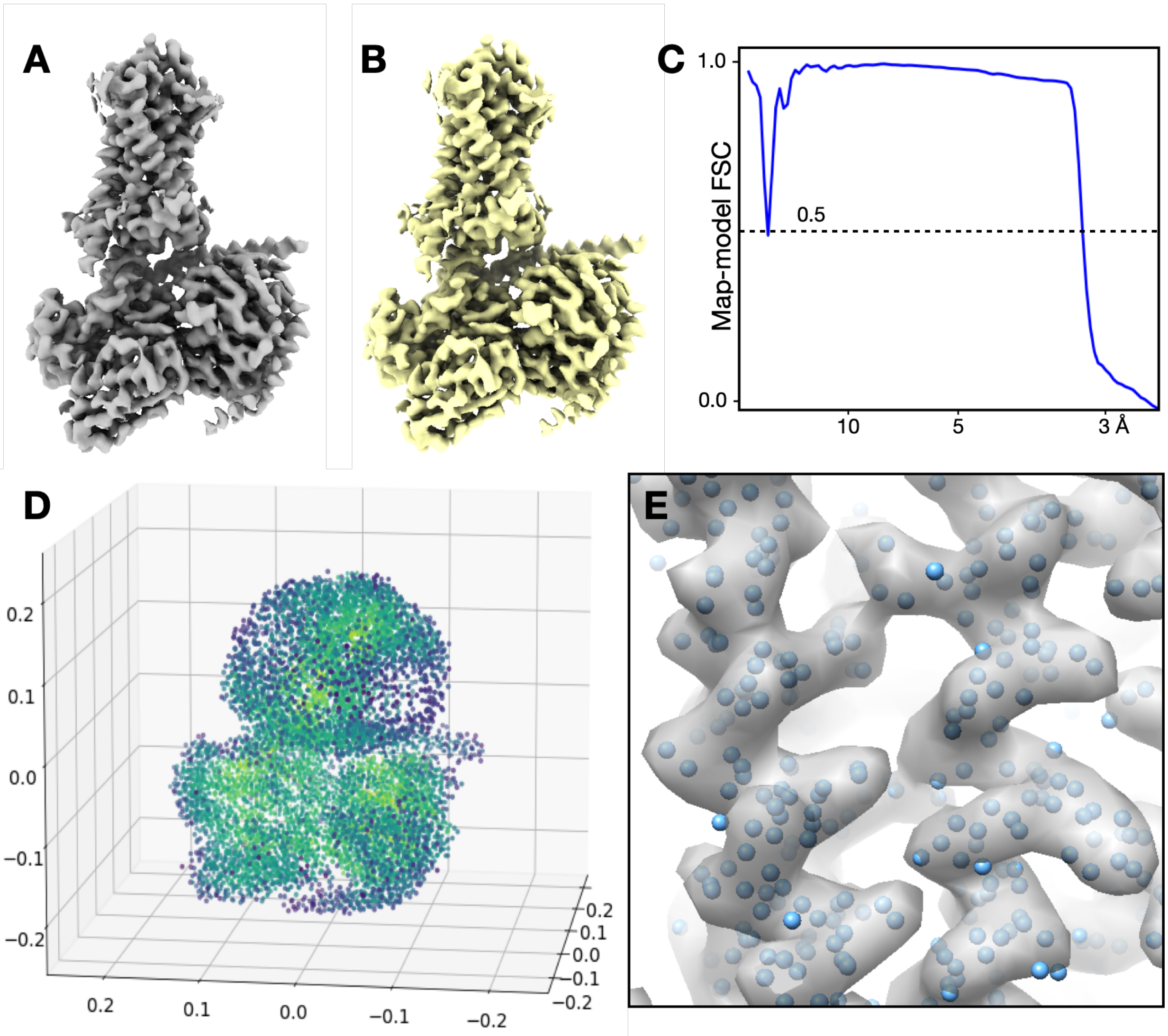

Fig S5. Visualization of GMMs. (A) Reconstruction of the GPCR using the voxel map representation, determined at 3.3Å resolution. $6x10^6$ floating point values are required to represent the structure. (B) GMM representation of the A, shown in isosurface view. $5x10^4$ floating point values are used to represent the structure. (C) FSC curve between A and B. The two structures are virtually identical up to 3.3Å. (D) Visualization of the GMM from B using 3D scatter plot. Each point is colored by the amplitude of the Gaussian function and the size of the points correspond to the width of Gaussian functions. (E) Overlay of the coordinates of Gaussian functions in the corresponding density map.

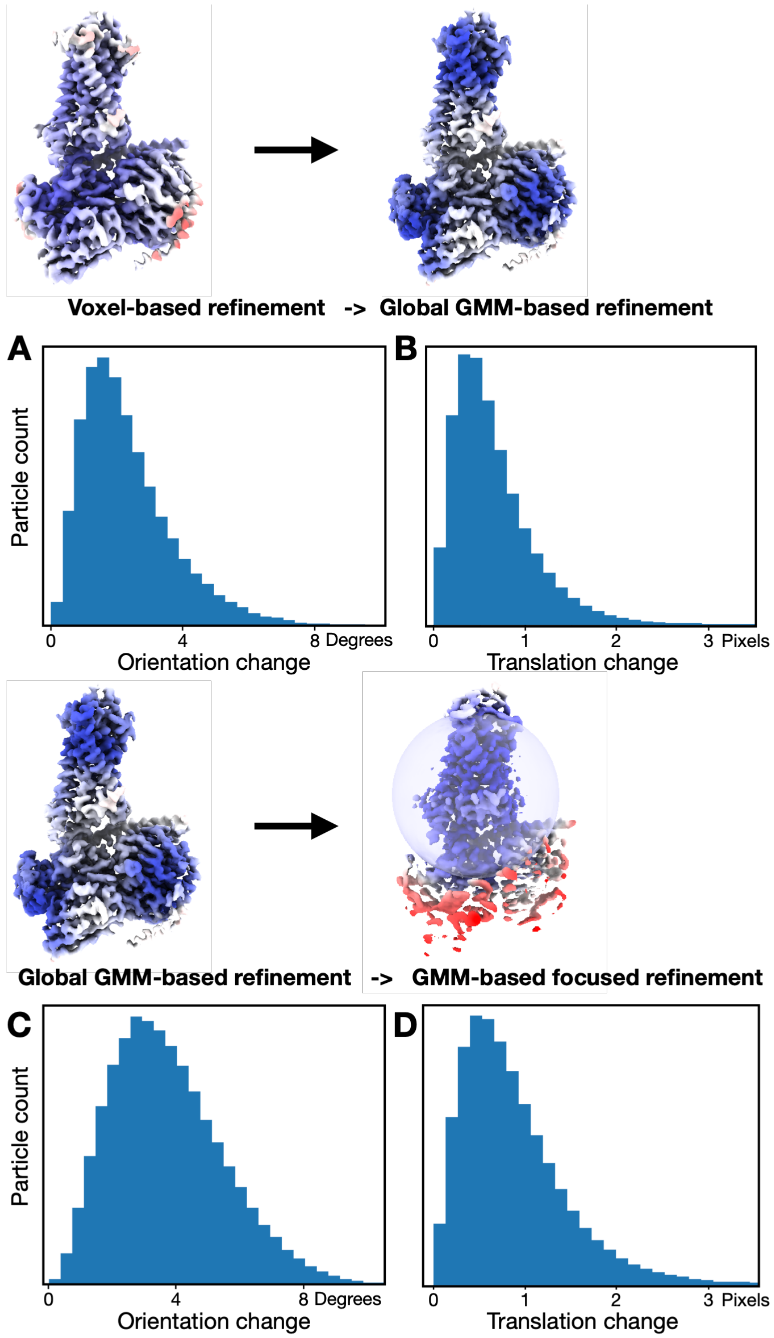

Fig S6. Orientation and translation change before and after GMM-based refinement. (A) Histogram of particle orientation assignment change after the GMM-based global refinement. Mean=2.34, std=1.37 degrees. (B) Histogram of particle translation change after the GMM-based global refinement. Mean=0.66, std=0.46 pixels. (C) Histogram of particle orientation assignment change after the GMM-based focused refinement. Mean=3.74, std=1.76 degrees. (D) Histogram of particle translation change after the GMM-based global refinement. Mean=0.89, std=0.62 pixels.

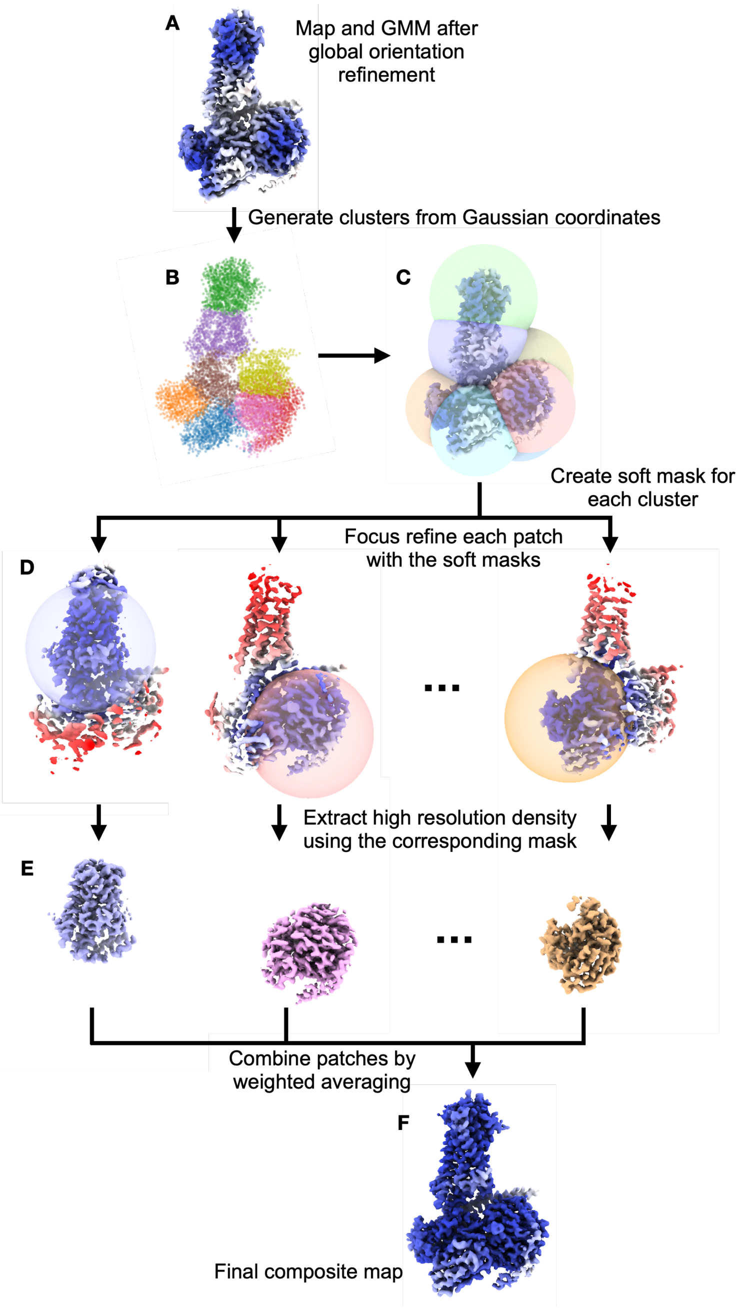

Fig S7. Diagram for the patch-by-patch refinement. (A) Input density maps and GMMs from the global orientation refinement. (B) Scatter plot of Gaussian coordinates, colored by clustering result. (C) Soft masks for each cluster/patch. Each mask is a sphere covering all Gaussian coordinates of the corresponding cluster, with a soft falloff. (D) Focused refinement results using the soft masks, colored by local resolution. Note that the same refinement process is done independently for the even/odd subsets of particles using the corresponding half map/GMM as reference. (E) Masked out density from the individual focused refinement result. (F) Final composite map generated by weighted averaging.

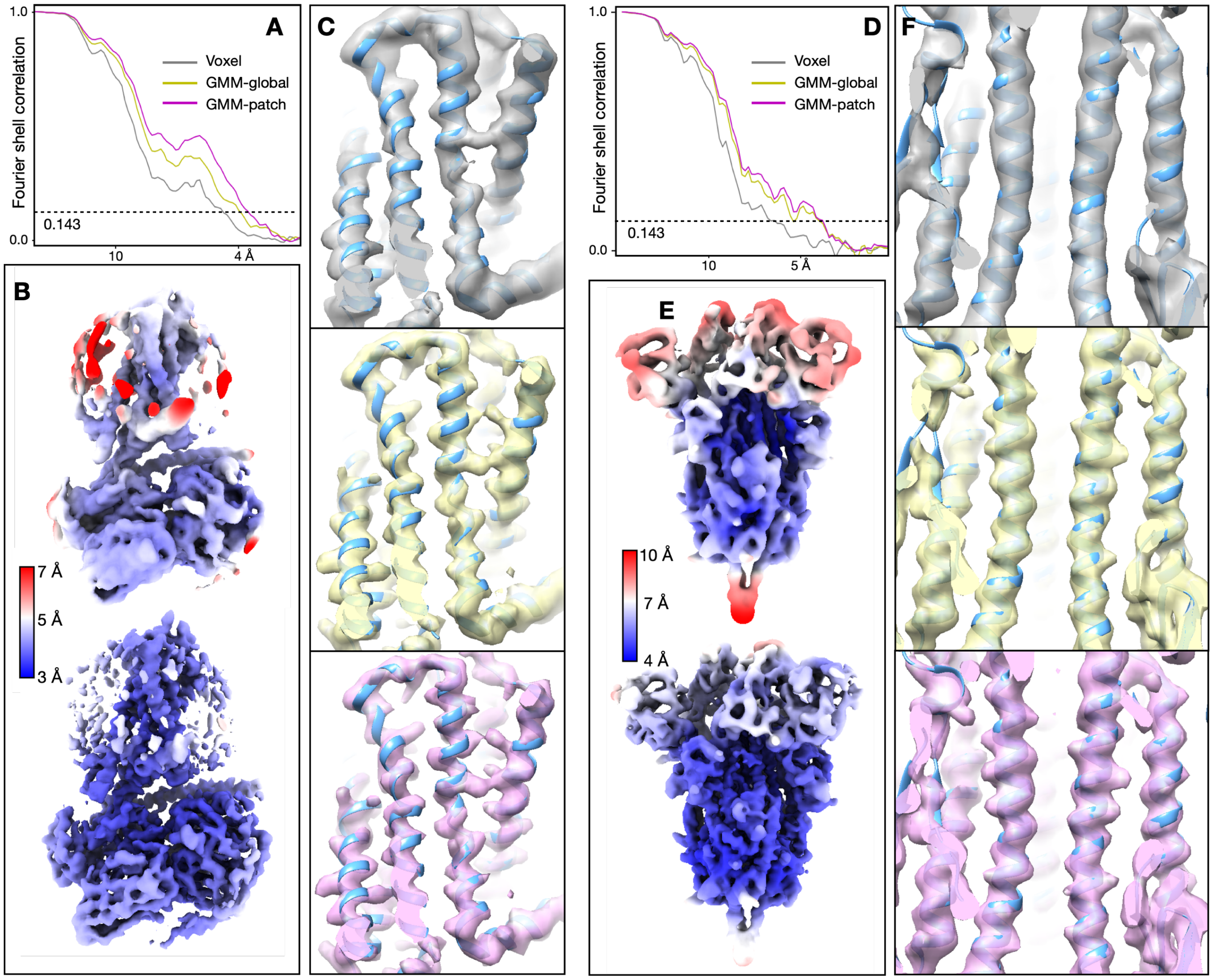

Fig S8. Performance of GMM-based refinement in datasets at lower resolution. (A-C) Refinement of a small subset of the GPCR dataset. (D-F) Refinement of a small subset of the SARS-COV2 dataset. (A, D) "Gold-standard" FSC curves of the reconstruction using voxel-based refinement (gray), global GMM-based refinement (yellow) and patch-by-patch refinement (pink). (B, E) Overall structure of the initial reconstruction and the final patch-by-patch refinement result, colored by local resolution. (C, F) Comparison of local features from the voxel-based, global GMM-based and patch-by-patch refinement.

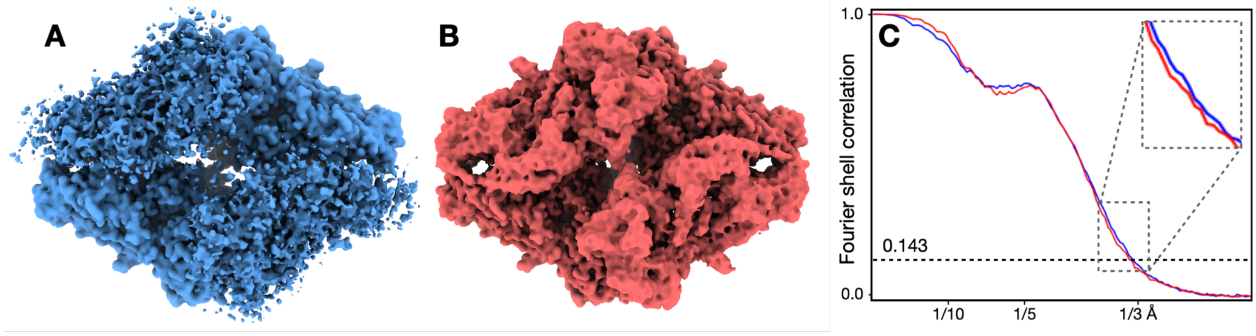

Fig S9. Impact of flexible domains on the global FSC curve. (A) Simulated density map of β-galactosidase, with two rigid subunits at 2.5Å and two flexible ones resolved at 15Å. (B) Same simulated map as A, with the two flexible subunits resolved at 7Å. (C) Comparison of "gold-standard" FSC curves of the two structures. Blue - A, red - B.

Supplementary movie 1

Structure comparison of the GPCR dataset (EMPIAR-10786). Gray: reconstruction using initial orientation. Pink: reconstruction after GMM-based patch-by-patch refinement.

Supplementary movie 2

Structure comparison of the SARS-COV2 dataset (EMPIAR-10492). Gray: reconstruction using initial orientation. Pink: reconstruction after GMM-based patch-by-patch refinement.

Supplementary movie 3

Structure comparison of the ABC transporter dataset (EMPIAR-10374). Gray: reconstruction using initial orientation. Pink: reconstruction after GMM-based patch-by-patch refinement.

Supplementary movie 4

Converting structural heterogeneity to particle orientation focusing on the Fab of the ABC transporter (EMPIAR-10374).
